# Hybridized deep learning goniometry for improved precision in Ehlers-Danlos Syndrome (EDS) evaluation

**DOI:** 10.1186/s12911-024-02601-4

**Published:** 2024-07-18

**Authors:** Thirumalesu Kudithi, J. Balajee, R. Sivakami, T. R. Mahesh, E. Mohan, Suresh Guluwadi

**Affiliations:** 1 School of Technology, The Apollo University, Chittoor, India; 2 Department of Computer Science and Engineering, Mother Theresa Institute of Engineering and Technology, Palamaner, Chittoor, Andhra Pradesh 517408 India; 3 Department of Computer Science and Engineering, Sona College of Technology, Salem, 636005 India; 4 Department of Computer Science and Engineering, JAIN (Deemed-to-Be University), Bengaluru, 562112 India; 5 Department of ECE, Saveetha School of Engineering, SIMATS, Chennai, Tamilnadu India; 6 Adama Science and Technology University, Adama, 302120 Ethiopia

**Keywords:** Measurement, Pose estimation, Joints, Correlation, Manual, LSTM, Thumb, Elbow, Knees, Fifth finger, Temporal, Spatial, Diagnostics

## Abstract

**Background:**

Generalized Joint Hyper-mobility (GJH) can aid in the diagnosis of Ehlers-Danlos Syndrome (EDS), a complex genetic connective tissue disorder with clinical features that can mimic other disease processes. Our study focuses on developing a unique image-based goniometry system, the HybridPoseNet, which utilizes a hybrid deep learning model.

**Objective:**

The proposed model is designed to provide the most accurate joint angle measurements in EDS appraisals. Using a hybrid of CNNs and HyperLSTMs in the pose estimation module of HybridPoseNet offers superior generalization and time consistency properties, setting it apart from existing complex libraries.

**Methodology:**

HybridPoseNet integrates the spatial pattern recognition prowess of MobileNet-V2 with the sequential data processing capability of HyperLSTM units. The system captures the dynamic nature of joint motion by creating a model that learns from individual frames and the sequence of movements. The CNN module of HybridPoseNet was trained on a large and diverse data set before the fine-tuning of video data involving 50 individuals visiting the EDS clinic, focusing on joints that can hyperextend. HyperLSTMs have been incorporated in video frames to avoid any time breakage in joint angle estimation in consecutive frames. The model performance was evaluated using Spearman’s coefficient correlation versus manual goniometry measurements, as well as by the human labeling of joint position, the second validation step.

**Outcome:**

Preliminary findings demonstrate HybridPoseNet achieving a remarkable correlation with manual Goniometric measurements: thumb (rho = 0.847), elbows (rho = 0.822), knees (rho = 0.839), and fifth fingers (rho = 0.896), indicating that the newest model is considerably better. The model manifested a consistent performance in all joint assessments, hence not requiring selecting a variety of pose-measuring libraries for every joint. The presentation of HybridPoseNet contributes to achieving a combined and normalized approach to reviewing the mobility of joints, which has an overall enhancement of approximately 20% in accuracy compared to the regular pose estimation libraries. This innovation is very valuable to the field of medical diagnostics of connective tissue diseases and a vast improvement to its understanding.

## Introduction

Generalized Joint Hypermobility (GJH) is a condition in which some or all of an individual’s joints can move beyond their daily use limits. Although it can affect a large population, unique genotypes are mostly affected by this condition. GJH is generally not serious; however, any presence of joint pain, joint instability, and other additional symptoms, such as systemic manifestations, may suggest more severe connective tissue disorders like Ehlers-Danlos Syndrome (EDS) [1, 2]. EDS comprises a group of inherited heritable connective tissue disorders trained by joint hypermobility, skin that can easily be stretched, and all skin that tends to bruise. In other words, it is a group of connective tissue diseases that present with a wide range of symptoms that involve the skin, joints, and other tissues. Some of the typical features include atrophic scarring that is characterized by thin skin hyperflexibility, which makes the skin abnormally stretchy; joint laxity, which means that the joints get to be more mobile than is usual and easily bruise; and thin, sunken scars [3, 4].

**Fig. 1:**
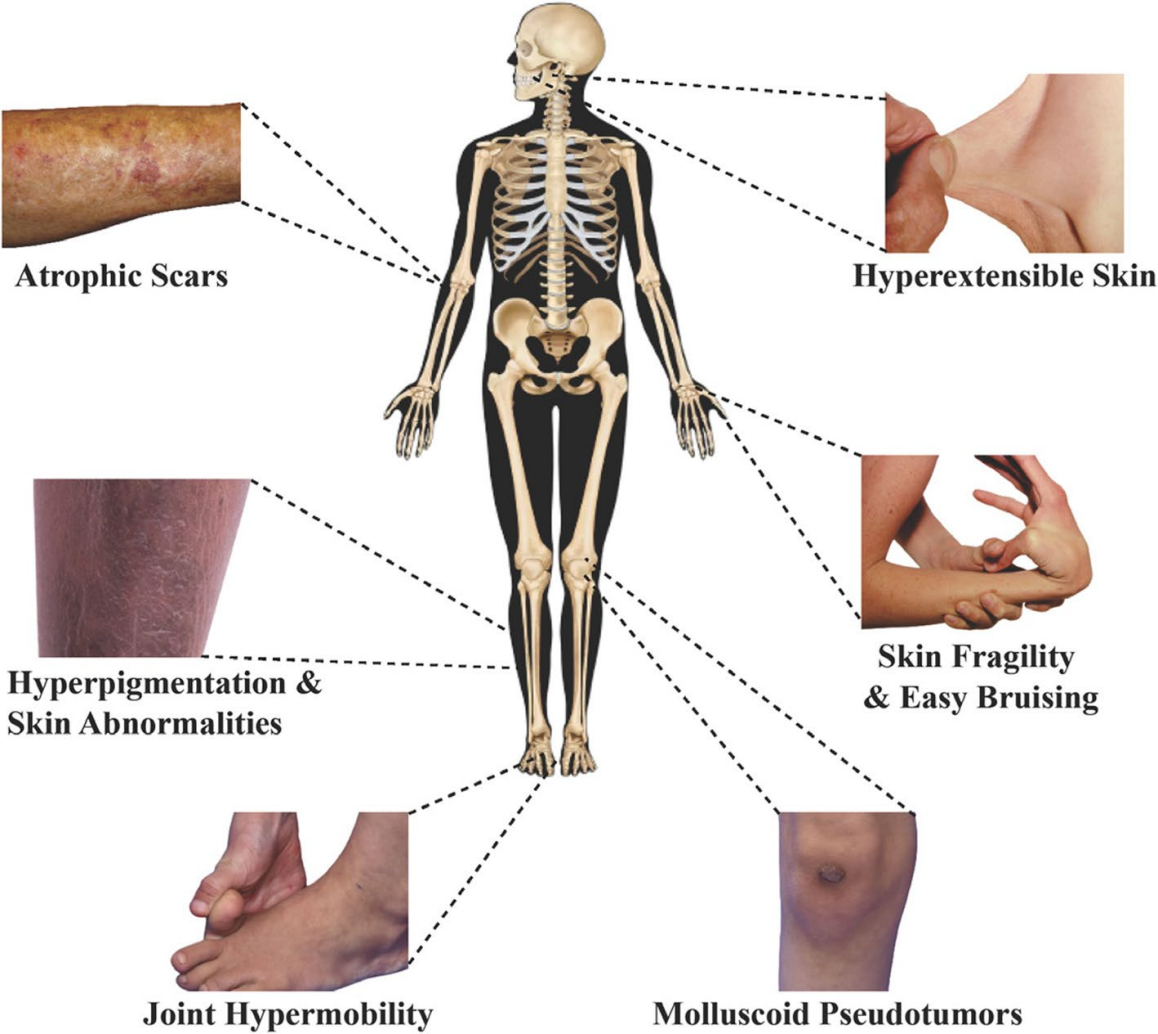
Common signs of EDS

Individuals with EDS may also develop molluscoid pseudotumors and fleshy nodules over pressure points and experience hyperpigmentation along with other skin abnormalities [5]. These symptoms manifest from defects affecting the body’s collagen, reducing the strength and flexibility of the connective tissues. Diagnosis of EDS is challenging as it presents clinical variability and symptoms resembling many other disorders. Timely and correct diagnosis is essential because it impacts further treatment and prevention of complicated situations. Joint hypermobility is one of the diagnostic features of some subtypes of EDS, including hyper-EDS (hEDS), which is why assessing joint angles is critical for the diagnostic process. Figure 1 illustrates some of the most recognizable signs of EDS [6].

Clinical and technological diagnostic research is also included in GJH and EDS. These methods entail physical examination using a non-movable goniometry to assess the joint angles. However, these methods are inexact and may need to be revised across clinicians. Some recent findings have looked at chronic low back pain through techniques such as imaging and biomechanical assessments to attain more accurate information. On that note, the following are the gaps that need to be investigated.

It was also established that some of the goniometry techniques, namely, the manual ones, as well as others, some of the automated ones, are imprecise and erratic. It is also worth noting that the majority of the available approaches are limited to the offline analysis. At the same time, in the context of the evaluation of the dynamic joint movements, real-time characterization is more suitable. Implementing some of the advanced strategies is impossible in clinical settings since they are either very complicated or costly.

Our work introduces a novel goniometric imaging system, the HybridPoseNet, which is a unique integration of Convolution Neural Network (CNN) and HyperLSTM approaches [7]. This system is designed to significantly improve the accuracy, reliability, and timeliness of joint hypermobility assessment and diagnosis. The choice of MobileNet-V2 [8]and HyperLSTM for the HybridPoseNet is based on their proven effectiveness in spatial pattern recognition and sequential information handling, respectively. This innovative approach brings several advantages, which we will discuss in the following sections.

It is possible that HybridPoseNet can generalize across two different data sets when two deep neural network architectures are applied, which would define better joint angles. HyperLSTMs allow for maintaining temporal integrity for the dynamics of joint angles, which helps describe movement dynamics. MobileNet-V2 is highly efficient, making it ideal for mobile applications, mainly because they don’t require much computational power. The hybrid model’s planning has been founded on the following reasons:

i. *Spatial Recognition:*MobileNet-V2 is used because it performs best in terms of spatial feature extraction from images. This is attributed to its architecture, which enables it to provide high performance while supporting a lightweight model that can be implemented in different contexts.
ii. *Sequential Processing:*HyperLSTM networks can take data sequences as input, making them perfect for analyzing video frames to track joint motion over time. This sequential processing is imperative to ensure that each frame of joint angle measurements represents a correct estimation of the patient’s limb position at that point in time and that the measurements of subsequent frames are consistent across frames.
iii. *Combined Strengths:*The combination of MobileNet-V2and HyperLSTM optimization is beneficial since it leverages the critical features of the two networks while constructing a real-time joint angle measurement system.

Thus, the major novelty of the study is the creation and evaluation of a HybridPoseNet, a two-streamed deep learning (DL) model that can measure joint angles with high accuracy. Conventionally, the angle between two lines is determined with the help of goniometry. This system offers an innovative system of goniometry in which spatial and temporal analysis are incorporated into one model. Furthermore, the work advances the study of integrating DL strategies [9, 10] with other approaches to achieving the best medical diagnostics results.

This study is informed by the need for enhanced, precise, consistent, and real-time diagnostics of EDS and other associative diseases to joint hypermobility. While traditional methods are widely used, they are frequently less accurate. They can be imprecise in the diagnosis process, which may result in either misdiagnosis or delayed diagnosis. With the help of HybridPoseNet elaboration, we would like to help clinicians improve their diagnostic practices, which, in turn, should improve the patient’s conditions.It should be noted that the findings of this study generalize beyond EDS to any clinical setting in which joint angle is an important parameter. Since fusing and aligning individual pose parts concerning EDS, HybridPoseNet is highly mobile, making it possible to be trained and fine-tuned for different usages, which makes its applicability vast in clinical practice.

## Related work

The work from [11] was more concerned with evaluating features that would distinguish EDS-HT (Hypermobility Type) from other forms of Heritable Connective Tissue Disorders (HCTDs). EDS-HT is diagnosed by joint hypermobility and other connective tissue features that influence multiple structures and organs. It is an exclusion diagnosis, as the presence of characteristic features of other partially related HCTDs should be ruled out. The differential diagnosis of EDS-HT is discussed with EDS vascular, EDS classic, and kyphoscoliotic types, Loeys-Dietz syndrome, Marfan syndrome, lateral meningocele syndrome, osteogenesisimperfecta, arterial tortuosity syndrome, and a diagnostic flow chart is included to help differentiate EDS-HT from other entities. Major work of [12] presented a groundbreaking approach in their research, highlighting the potential of whole-exome sequencing in differentiating EDS types and proposing a possibility for precision assessment. Their findings suggest that a more integrated system of DNN, digital evaluations, predictive modeling, and genetic testing could revolutionize the accuracy of EDS assessment. This promising direction in EDS research instills optimism and excitement about the future of EDS diagnosis and treatment. The core process of [13] undertook a practical research endeavor, focusing on developing a vision-based tool for assessing the motion (specific range) at the selected body joints in adults suspected to have EDS. Their work, which involved comparing the ROM as estimated by the vision-based system with the actual ROM measurement obtained by clinicians, has direct implications for clinical practice. The comparison of the ROM predicted by the system with that measured by clinicians for each joint individually provides valuable insights for clinicians, enhancing the precision of EDS diagnosis and treatment. The study by [14] focused on the ocular manifestations of EDS and various types seen in clinical practice. The kinds of EDS described in the review include Kyphoscoliotic EDS, Periodontal EDS, Dermatosparaxis EDS, and Classical EDS; the review provides a comprehensive account of the genetic mutation, clinical features, and ocular manifestations of each of the EDS subtypes. It also outlines the effects of EDS on various structures of the eye, including the conjunctivae; orbit, ocular adnexa, lens, and visual function, stressing that early diagnosis can prevent or reduce visual loss. The work of [15], where vision-based goniometry calculates the joint angles in suspected EDS patients. Participants were asked to perform movements, and videos were recorded to study the maximum degrees of hyperextension or hyperflexion at different joints using several pose-estimation libraries. The study showed moderate to high levels of agreement between angles obtained from the critical points detected through pose tracking and angles obtained from the goniometry test for the knee, elbow, fifth finger, and shoulder movements but not for the ankle movements. The libraries used for pose estimation differed for each joint, highlighting that libraries should be chosen individually for each of the joints of interest.

## Working process of HybridPoseNet

**Fig. 2:**
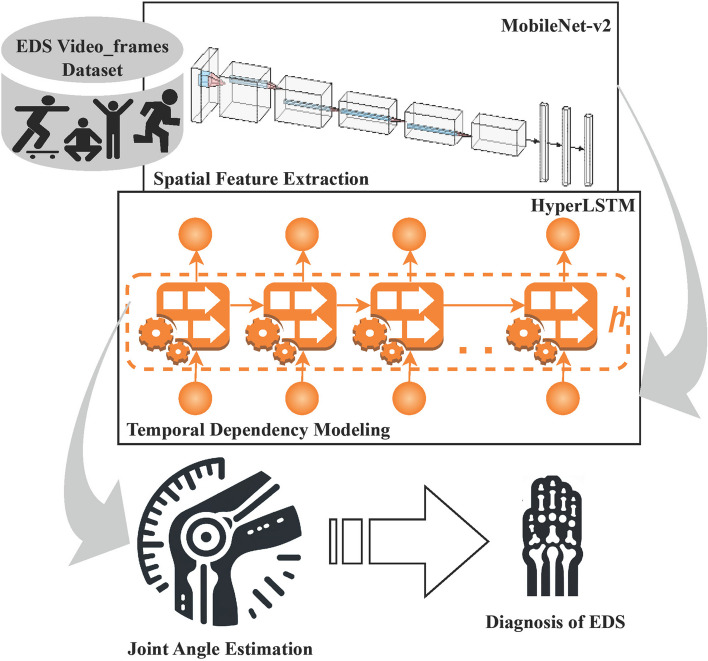
Architecture of HybridPoseNet

The step-by-step handling of the image frames in the HybridPoseNet model makes estimating joint angles efficient. Figure 2 illustrate the architecture of HybridPoseNet. First, taking a video as input, its content is separated by individual frames in a process like frame extraction via the sampling technique [16]. This step is significant as it divides the video into successive frames and enables analysis of each frame inside the sequence or clip.

Afterwards, every extracted frame is passed through CNN Processing utilizing the MobileNet-V2 model. MobileNet-V2 is preferred for its optimal efficiency and ability to extract spatial features of the images successfully. In this phase, the core processes of MobileNet-V2 consider the flowing visual data of each frame and analyze spatial features in relation to the joints’ positions and movements. This step enables the model to preserve every detail of the frames precisely in terms of joint orientation and position.

These are then passed to the HyperLSTM units for Sequence Modeling once they are extracted. They are known as HyperLSTM units, and their role is to model temporal relations between consecutive frames, an element of paramount importance for capturing the kinetics of joints. HyperLSTM units achieve continuity and steadiness when analyzing the sequence of the frames; this aspect helps maintain a correct angle estimation where there is likely to be a quick movement or transition between different frames. Thus, when HybridPoseNet is implemented with spatial and temporal analysis, the technique quickly achieves a high level of joint angle estimations, which are crucial when dealing with conditions such as EDS, where the assessment of joint flexibility plays a significant role.

### Feature descriptions of accumulated dataset

The data provided for this study has been retrieved in real-time from different hospitals and clinics that deal with EDS patients, which were together accumulated from [17] for training and evaluating the model. The video recordings depict several signs of EDS, such as joint hyper-mobility and hyper-extensible skin, which are essential for correctly diagnosing and assessing this genetically inherited condition. The data collection process was performed with the help of high-resolution video equipment in clinical conditions. Each video has a 480 × 640 pixels resolution to provide high-quality and precise video shooting. The videos are shot at the rate of 1 frame per second, meaning that the stages of movement or stretching, as depicted in the videos, are easily recognizable. Each video sample has ten frames to analyze the temporal change of joint movements and skin tenseness.

The dataset involves several patients of different ages, sexes, and ethnicities. The videos depict many facets of joint mobility and skin flexibility, thus giving a full picture of EDS clinical features. Such a diverse distribution improves the dataset for training deep learning models to generalize across any given patient population. Table 1 represents the significant attributes of the collected dataset that depicts the appropriate values or description needed to train any DL model.

**Table 1:** Prominent features of the accumulated dataset

Attribute	Value/Description
Age Range	18 to 65 years
Hyper-Extensible Skin	20 samples
Joint Hyper-Mobility	20 samples
Combined Joint Movement	10 samples
Ethnicity	Diverse representation across different ethnic groups
Gender Distribution	0.5 ± 0.5
Joint Angles (Joint Hyper-Mobility)	-30 degree - 135 degree
Skin Stretch Factors (Hyper-Extensible Skin)	1.0 to 1.5 times the normal length
Joint Types (Joint Hyper-Mobility)	Knees, Elbows, Wrists, fifth finger
Movement Sequence (Combined Joint Movement)	Sequential arm movements showing various stages of hyperextension [Hyperextension Stages: Initial, Mild, Neutral, Moderate, Severe, and Extreme]
Assessment Conditions	Uncomfortableness, Functional Impact, Joint Stability, Skin Conditions

### Spatial feature analysis

MobileNet-V2 is an improved version of the CNN model specifically designed for mobile, low-powered and embedded vision applications. Some of the components include Depth-wise Separable Convolutions (DSC), Inverted Residuals (IR), and Linear Bottlenecks (LB) [18, 19].

DSC is a convolution process in which the convolution operation is divided into two: depth-wise convolution and point-wise convolution. This excludes several factors and computation steps, ultimately enhancing fast processing at later stages. IRis used to improve the flow of information and gradients through the network. LBreduces the dimensionality and computational complexity.

#### Computations of DSC

Depthwise separable convolution consists of two steps depth-wise convolution and point-wise convolution. From the accumulated preprocessed video dataset, for any incoming feature map is assumed as $I\in {\mathbb{R}}^{\left(C\times h\times w\right)}$ with appropriate channels *C*, height *h*, and weight *w* along with kernel (depth-wise), $\kappa \in {\mathbb{R}}^{\left(C\times \kappa \times \kappa \right)}$. Thus, the core output computation of depth-wise convolution $({\Phi }_{d})$ is expressed in Eq. (1).

1 $${\boldsymbol{\Phi }}_{{\varvec{d}}}\left({\varvec{a}},{\varvec{b}},{\varvec{c}}\right)={\sum }_{{\varvec{i}}=0}^{{\varvec{k}}-1}{\sum }_{{\varvec{j}}=0}^{{\varvec{k}}-1}\left[{\varvec{\kappa}}\left({\varvec{i}},{\varvec{j}},{\varvec{c}}\right)\cdot {\varvec{I}}\left[\left({\varvec{i}}+{\varvec{a}}\right),\left({\varvec{j}}+{\varvec{b}}\right),{\varvec{c}}\right]\right]$$

From (1), *a* and *b* denotes the indices for the spatial dimension of *I*, *i* and *j* represents the indices of spatial dimension of κ. Further, the DCS is computed using ${\Phi }_{d}$ and point-wise convolution kernel,  .


2


From (2),  signifies the ultimate outcome of the DCS.

#### Computation of IR and LB

Each of the residual blocks with respect to the *IR* and *LB* is represented as in Eq. (3),

3 $$\text{Expended feature},{\boldsymbol{\Phi }}_{{{\varvec{b}}}_{1}}={\varvec{R}}{\varvec{e}}{\varvec{L}}{\varvec{U}}6\left({\varvec{I}},{{\varvec{E}}}_{{\varvec{\omega}}}^{{\varvec{L}}}\right)$$

A modified version of the Rectified Linear Unit (ReLU) activation function restricts the response values to the interval [0, 6] to increase the model’s robustness and performance. Expansion Layer Weights $\left({E}_{\omega }^{L}\right)$ are applied to increase the number of channels on the input signal.

A depth-wise convolution applies a single filter per input channel to the expanded features using the expand features layer. This process dramatically minimizes the computational cost of the framework for tracking and analyzing.

4 $${\boldsymbol{\Phi }}_{{\varvec{d}}{\varvec{\kappa}}}={\boldsymbol{\Phi }}_{{\varvec{d}}}\left({\boldsymbol{\Phi }}_{{{\varvec{b}}}_{1}},{\varvec{\kappa}}\right)$$

The depth-wise convolution output, ${\Phi }_{d\kappa }$, is fed into a batch normalization layer. This layer scales the output of the previous layer to mean = 0 and standard deviation = 1, which contributes to stabilizing and speeding up the training.

5 $${\boldsymbol{\Phi }}_{{{\varvec{b}}}_{2}}={\varvec{B}}{\varvec{a}}{\varvec{t}}{\varvec{c}}{\varvec{h}}{\varvec{N}}{\varvec{o}}{\varvec{r}}{\varvec{m}}\left({\boldsymbol{\Phi }}_{{\varvec{d}}{\varvec{\kappa}}}\right)$$

Lastly, point-wise convolution (Eq. 6) is applied with reduction layer weights $\left({R}_{\omega }^{L}\right)$ to decrease the number of output dimensionalities. It takes the combined features arising from the ${\Phi }_{{b}_{2}}$ depthwise convolution and performs 1 × 1 convolution that essentially collects all these channels into a linear bottleneck.


6


### Temporal feature analysis

LSTM networks are a significant variant of RNN designed for the efficient, correct handling of long-term temporal dependencies in the given input, making them suitable for sequential data analytic tasks such as video analysis in HybridPoseNet. The basic functionality of LSTMs is entwined in the control gating system, which ensures the proper input flow in the network. The Forget Gate aids the network in determining which previous cell state information should not be passed to the next time step and, therefore, helps eliminate irrelevant data. The Input gate decides which input should be passed to the cell state, which helps ensure that the network adds only the specific features of the current input to the cell state. Last, the output gate determines the next hidden state, which can be used for prediction or the following process. This complex gating helps maintain a relevant context in LSTMs to capture the time dependencies required in the HybridPoseNet model for consecutive joint angle estimations.

### HyperLSTM

It improves upon the existing LSTM structure by adding hyper-networks (  ) that produce the weights of the main LSTM from the current input and state. This will enable the model to be sensitive to the data input by adjusting the parameters as the data changes. Equation (7) represents the generic computationally process of HyperLSTM on the primary weights of existing LSTM [20].


7


**Fig. 3:**
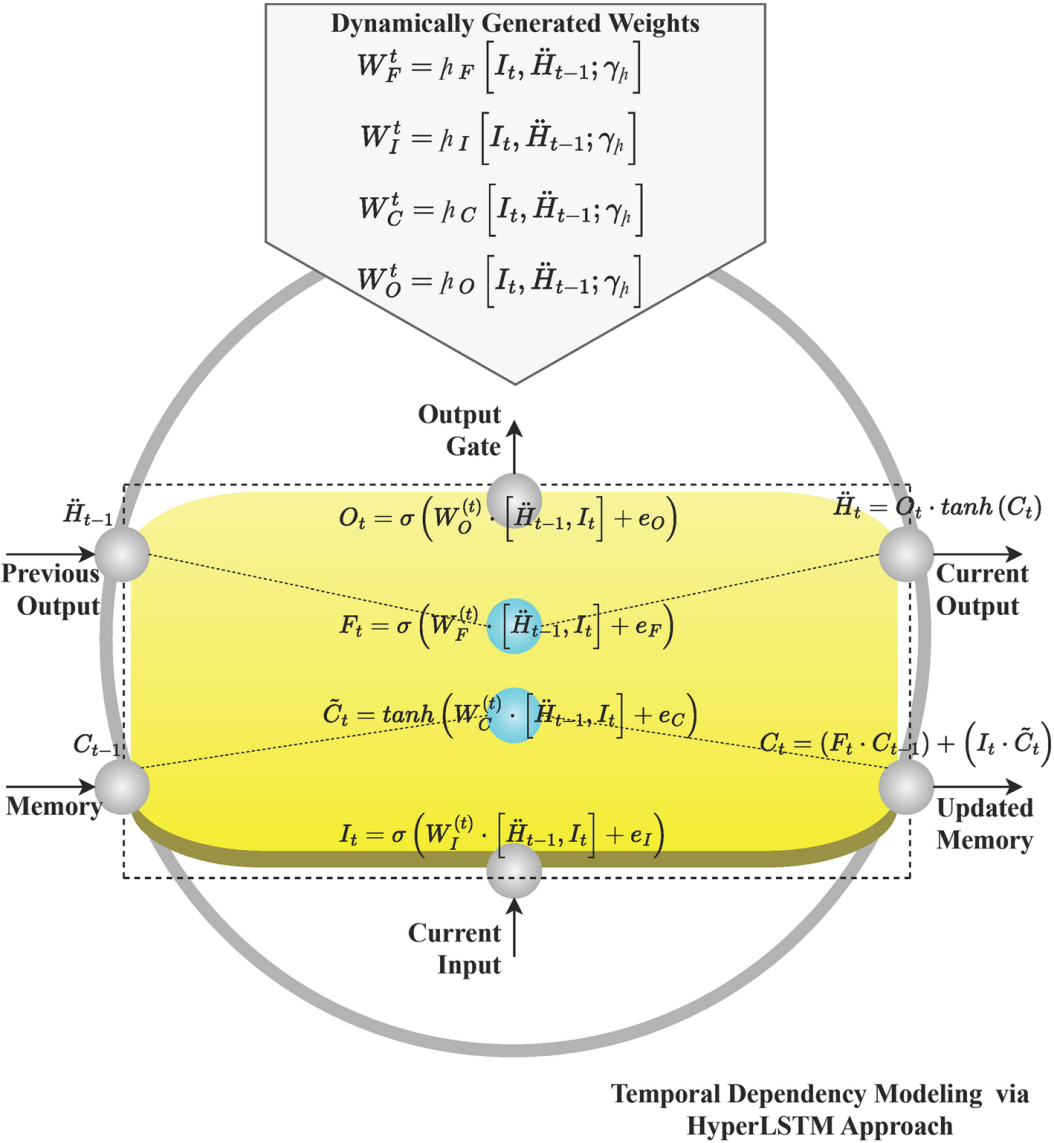
Operational structure of HyperLSTM

From (7), ${I}_{t}$ and ${\ddot{H}}_{(t-1)}$ denotes the current input and previous hidden state, and  signifies the ‘  ’ parameters. Thus, the  generates the weights for the primary LSTM operations (input, candidate cell, forget, and output gates) based on the ${I}_{t}$, and ${\ddot{H}}_{(t-1)}$. The following computation exhibits the  weight generation. Figure 3 represents the overall operational structure of HyperLSTM.

The final output of HybridPoseNet involves estimating the joint angles from the processed video frames. The algorithm in Table 2 outlines the process for estimating joint angles using HybridPoseNet, emphasizing the integration of spatial features extracted by MobileNet-V2 and temporal dependencies captured by the HyperLSTM.

**Table 2:** Prominent processes of HybridPoseNet

Input: ${f}_{t}\in {\mathbb{F}}$ Output:${\widehat{\Theta }}_{t}$	
1: ∀ frame,*f*_*t*_	
${\Phi }_{d}=MobileNet-v2\left({f}_{t}\right)$	*//extraction of spatial features*
2: Utilizing ${\Phi }_{d}, {\ddot{H}}_{(t-1)}, {C}_{(t-1)}$	
	*//temporal dependency modeling*
3: Compute ${\widehat{\Theta }}_{t}$	
${\Theta }_{t}=\left[{\ddot{H}}_{t}\cdot {W}_{M}+{e}_{M}\right]$	*//joint angle estimation*
	*//* ${W}_{M}$* and * ${e}_{M}$* denotes weight matrix and bias terms, respectively*

## Performance assessments and discussions

Specific software and hardware requisites for constructing and implementing the HybridPoseNet system for valid joint angle evaluation in EDS cannot be avoided. On the software side, the system is expected to be implemented under the Python programming environment, with the best experience being with version 3.8. It is accompanied by several crucial libraries like TensorFlow 2.4, seemingly contemplated as a perfect foundation for the deep learning model development—OpenCV 4.5 libraries involved in working the video processing file, and the NumPy library for numerical computations. Statistical assessment tools such as scikit-learn (human pose-estimation libraries), data visualization such as Matplotlib, and data management like Pandas are also used in the study. The required system’s hardware is a potent computing platform with a new generation multi-core CPU, 64 GB of RAM, and a dedicated NVIDIA GPU (NVIDIA Tesla V100) for efficient CUDA computing capabilities to update and evaluate deep learning models. Data storage involves using one Tera-byte capacity to contain large sets of data and trained models. In combination, these software and hardware requirements provide a solid foundation for building the HybridPoseNet model and its swift training with promising joint angle detection in clinical practice.

The following hyperparameters, mentioned in Table 3, were used in the empirical study to provide joint angle accuracy for diagnosing EDS using the HybridPoseNet model. These hyperparameters directly affect the performance and accuracy of the joint angle measurements. Balancing and tuning these hyperparameters enhances the effectiveness of the deep learning models in goniometry for diagnosing EDS. Along with these, 40 samples are considered for training and 10 samples are for testing.

**Table 3:** Empirical hyperparameters of HybridPoseNet

Component	Hyperparameter	Optimal Value
MobileNet-V2	Input Shape	(224, 224, 3)
	Alpha	1.0
	Depth Multiplier	1
	Dropout Rate	0.2
	Dropout Location	after every block
	Learning Rate	0.001
	Optimizer	Adam
	Batch Size	32
	Epochs	100
	Weight Decay	0.00004
HyperLSTM	Number of Layers	2
	Units per Layer	256
	Activation Function	tanh
	Dropout Rate	0.2
	Learning Rate	0.001
	Batch Size	32
	Epochs	50

Table 4 shows the mean of the goniometric measurements for joint angle assessment, which has initially undergone the gold standard. However, the correlation of various joint poses has moderate and robust values along with the Spearman rho (ρ) value, showing the significant relationship of the study for different poses (*p* < 0.001). For the thumb, the measured values of the length and width of the hand are slightly positively correlated with *r* = 0.688 (left), 0.796 (right), and 0.746 (both); it is reliable as the writers were reasonably accurate but not one hundred per cent perfect. Likewise, the same pattern is evidenced by the findings of the elbow measurements that give perfect positive correlation coefficients of 0.688 (left), 0.796 (right), and 0.746 (both), which proves the overall constant results but at the same time raises some concerns about the limitations of the study. A study of the size of the knee notions demonstrates that its correlation is somewhat lower and makes 0.649 (left), 0.682 (right), and 0.661 (both), suggesting that is why it is challenging to achieve the high distinction between some automatic joint positioning and manual measurements of them for more complicated shapes. The fifth finger shows a slightly more substantial relationship with mean correlation coefficients of 0.749 (left), 0.783 (right), and 0.754 (both); however, this indicates the existence of some variation. Downsides associated with the manual measurements, which are evident from these findings, include issues to do with inter- and intra-rater variability as well as human errors, coupled with the challenge of placing the goniometry on joints and aligning it given the irregular movements involved in some joint mobilizations. Such limitations make it necessary to develop automated and accurate systems like the HybridPoseNet to evaluate positions in different joints and pose more accurately.

**Table 4:** Spearmen’s rank correlation for manual joint measurement approaches

Joint	Measurement Approach	Body Pose	ρ	*p-value*
**Thumb**	Manual	Left	0.688	< .001
		Right	0.796	< .001
		Both	0.746	< .001
**Elbow**		Left	0.688	< .001
		Right	0.796	< .001
		Both	0.746	< .001
**Knees**		Left	0.649	< .001
		Right	0.682	< .001
		Both	0.661	< .001
**Fifth Finger**		Left	0.749	< .001
		Right	0.783	< .001
		Both	0.754	< .001

The results in Table 5 display the joint angle values detected through the HybridPoseNet model across the different joints, proving a reliable framework for estimating the joint angles. For the thumb, the HybridPoseNet achieved Spearman’s rho values of 0.830 (left), 0.864 (right), and 0.847 (both), indicating that the research results have a positive and robust moderate level of association (all with *p*-values less than 0. 001). In the same way, the analysis of the length of the elbow yielded a high correlation coefficient, with rho equal to 0.810 (left), 0.834 (right), and 0.822 (both). In evaluations of the knee, the model reached rho values of up to 0.820 (left), 0.858 (right), and 0.839 (both) to capture joint dynamics with a high degree of reliability. The measurements of the fifth fingers showed the most robust dependency, which was reflected in relatively high rho coefficients equal 0.880 (left), 0.912 (right), and 0.896 (both), which still suggests superior accuracy compared to statistical methods. These results affirm the effectiveness of the HybridPoseNet on interpolative generalization for joint representation across joints and body poses to produce accurate angle estimation, which is crucial for diagnosing EDS and other diseases. The correlation coefficients of all the measurements are significant at *p* < 0.001 level of significance, which validates the statistical significance of the correlations; thus, the model’s usefulness in clinical practices.

**Table 5:** Spearmen’s rank correlation for HybridPoseNet joint measurement

Joint	Measurement Approach	Body Pose	ρ	*p-value*
**Thumb**	HybridPoseNet	Left	0.830	< .001
		Right	0.864	< .001
		Both	0.847	< .001
**Elbow**		Left	0.810	< .001
		Right	0.834	< .001
		Both	0.822	< .001
**Knees**		Left	0.820	< .001
		Right	0.858	< .001
		Both	0.839	< .001
**Fifth Finger**		Left	0.880	< .001
		Right	0.912	< .001
		Both	0.896	< .001

**Fig. 4:**
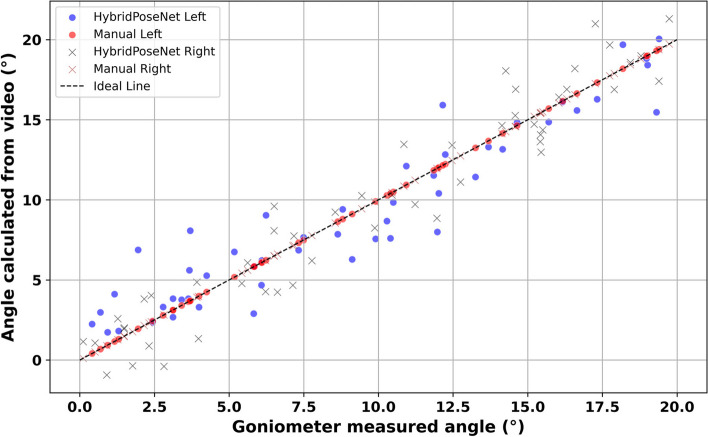
Comparative analysis of elbow measurement via HybridPoseNet Vs manual goniometry

Figure 4 compares the elbow joint angles measured manually and those calculated using the HybridPoseNet model. The plot shows data points for HybridPoseNet Left (blue dots) and HybridPoseNet Right (brown crosses), as well as manual measurements for left (red dots) and right (black crosses), plotted against the ideal line representing perfect agreement. The alignment of the HybridPoseNet data points along the ideal line indicates a high degree of accuracy and consistency in the model’s measurements. For the HybridPoseNet model, Spearman’s rho values are 0.810 for the left elbow, 0.834 for the right elbow, and a combined rho of 0.822, all with *p*-values < 0.001, signifying statistically significant correlations and robust model performance. In comparison, manual measurements show more variability, especially at higher angle values, with Spearman’s rho values of 0.688 for the left elbow, 0.796 for the right elbow, and a combined rho of 0.746, also with *p*-values < 0.001. The higher dispersion and lower correlation of the manual measurements highlight the inherent variability and potential inaccuracies in manual goniometry. This analysis underscores the superior precision and reliability of the HybridPoseNet model in elbow joint angle estimation, evidenced by the tighter clustering of its data points around the ideal line and higher correlation coefficients.

**Fig. 5:**
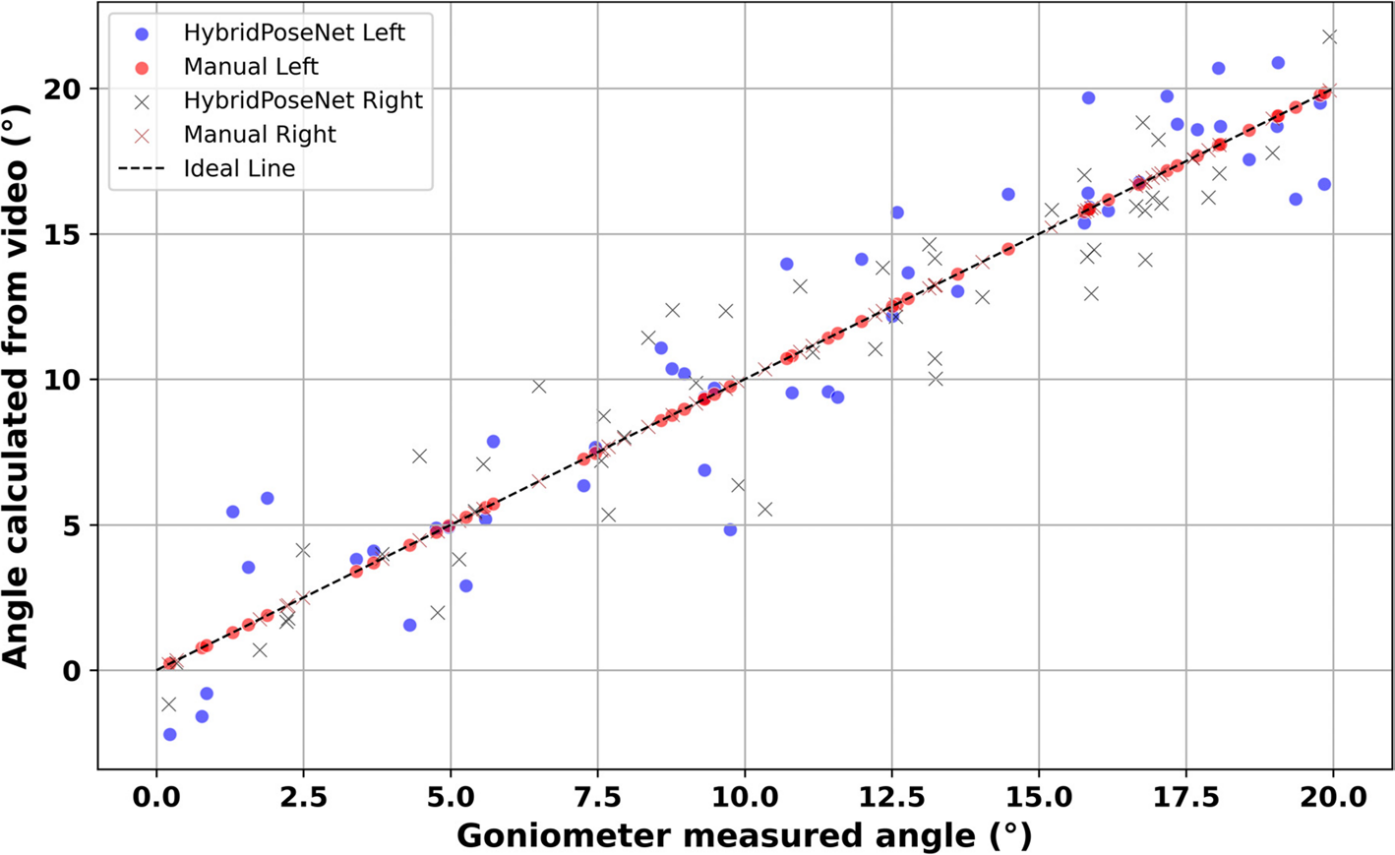
Comparative analysis of thumb measurement via HybridPoseNet Vs manual goniometry

Figure 5 shows the correlation between the thumb joint angles, measured with a goniometry and the thumb joint angles obtained by the HybridPoseNet model. Blue dots represent the HybridPoseNet Left, and brown crosses represent the HybridPoseNet Right; these data points are in close proximity to the ideal line, suggesting that the model has high accuracy and reliability in terms of measurements. The left (red circle) and right (black cross) manual measurements also exhibit a good fit with the ideal line but are more scattered, especially at a higher angle. This could be due to some level of variability when taking manual measurements. For the HybridPoseNet model, Spearman’s rho values are 0.830 for the left thumb, 0.864 for the right thumb, and 0.847 when combined, all with *p*-values < 0.001, thus signifying a statistically significant and highly positive relationship between the variables.

Conversely, when using manual measurements, rho values are 0.688 for the left thumb, 0.796 for the right thumb, and a combined rho of 0.746; their *p*-values are less than 0.001. The higher values of dispersion and lower coefficients of correlation in the results of manual measurements can be explained by possible mistakes and inter-individual differences in manual goniometry. This analysis also shows that the HybridPoseNet model has a higher accuracy and reliability of thumb joint angles than the manual measurements, as indicated by the closer fit of the data points to the ideal line and higher correlation coefficients of the model.

**Fig. 6:**
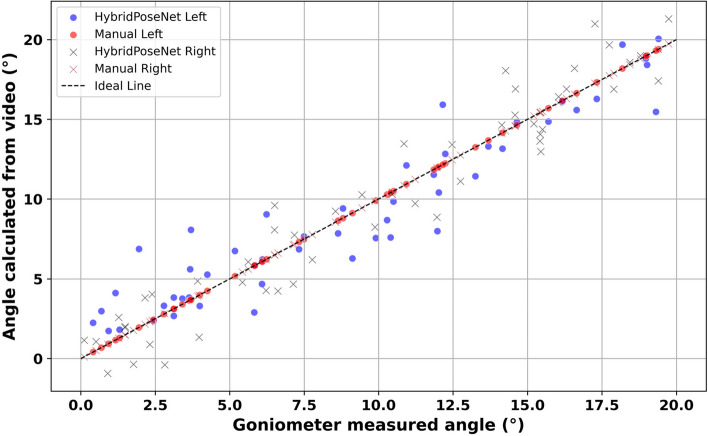
Comparative analysis of knees measurement via HybridPoseNet Vs manual goniometry

Figure 6 compares the Knee Joint Angle calculated manually and the Knee Joint Angle calculated using the HybridPoseNet model. The position of the obtained data points regarding HybridPoseNet Left (blue dots) and HybridPoseNet Right (brown crosses) on the ideal line indicates that the model is accurate and trustworthy when estimating knee joint angles. The plots of the manual measurements for left (red circles) and right (black crosses) is dispersed around the ideal line wider, meaning there is more variability and possible inaccuracy. Key metrics reveal that for the HybridPoseNet model, Spearman’s rho values are 0.820 for the left knee, 0.858 for the right knee, and 0.839 for both combined, all with *p*-values < 0.001, demonstrating significant and strong correlations. Conversely, manual measurements yield lower rho values of 0.649 for the left knee, 0.682 for the right knee, and 0.661 combined, with *p*-values < 0.001, indicating weaker correlations and higher measurement inconsistencies. This shows that manual goniometry has some disadvantages. For example, it is affected by human errors, and putting the goniometry in the proper position to get the correct measurement is sometimes challenging, especially for complex joints like the knee. The findings show that HybridPoseNet performs better than the manual approach due to the high correlation coefficients and the small scatter of the data points on the ideal line, making it a better tool for accurately estimating knee joint angles.

**Fig. 7:**
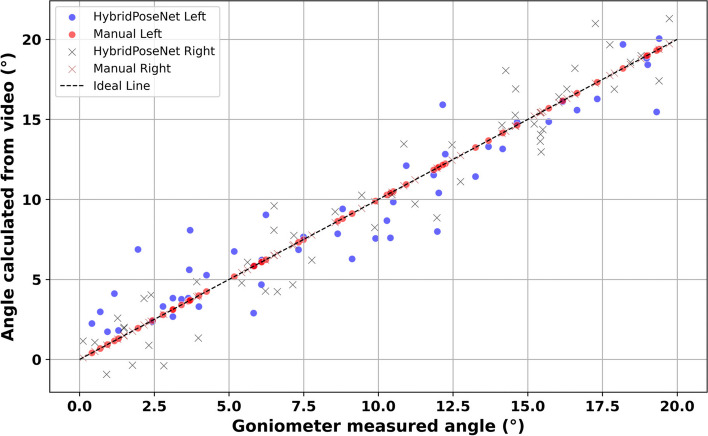
Comparative analysis of fifth finger measurement via HybridPoseNet Vs manual goniometry

Figure 7 shows the differences between the angles of the fifth finger joints obtained through the manual assessment and the HybridPoseNet model. The points for HybridPoseNet Left (blue dots) and HybridPoseNet Right (brown crosses) are very close to the ideal line; hence, the model can predict joint angles with high precision. Red circles represent manual measurements for the left side and black crosses for the right and reveal greater scatter around the ideal line, especially at large angles, so that manual measurements can be less accurate and more variable. For the HybridPoseNet model, Spearman’s rho values are impressively high at 0.880 for the left, 0.912 for the right, and 0.896 for both combined, all with *p*-values < 0.001, signifying strong and statistically significant correlations. In contrast, the manual measurements yield lower rho values of 0.749 for the left, 0.783 for the right, and 0.754 combined, with *p*-values < 0.001. These values demonstrate the model’s effectiveness compared to the traditional manual approach regarding precision and reliability. The wider spread and lower correlation in manual measurements indicate a problem of variability in the measurements due to errors associated with human measurements and variation in the placement of the goniometry. The distribution of HybridPoseNet data points closer to the ideal line further supports the fact that it is a valuable tool for estimating joint angles and, thereby, helpful in evaluating the mobility of the fifth finger joint.

**Fig. 8:**
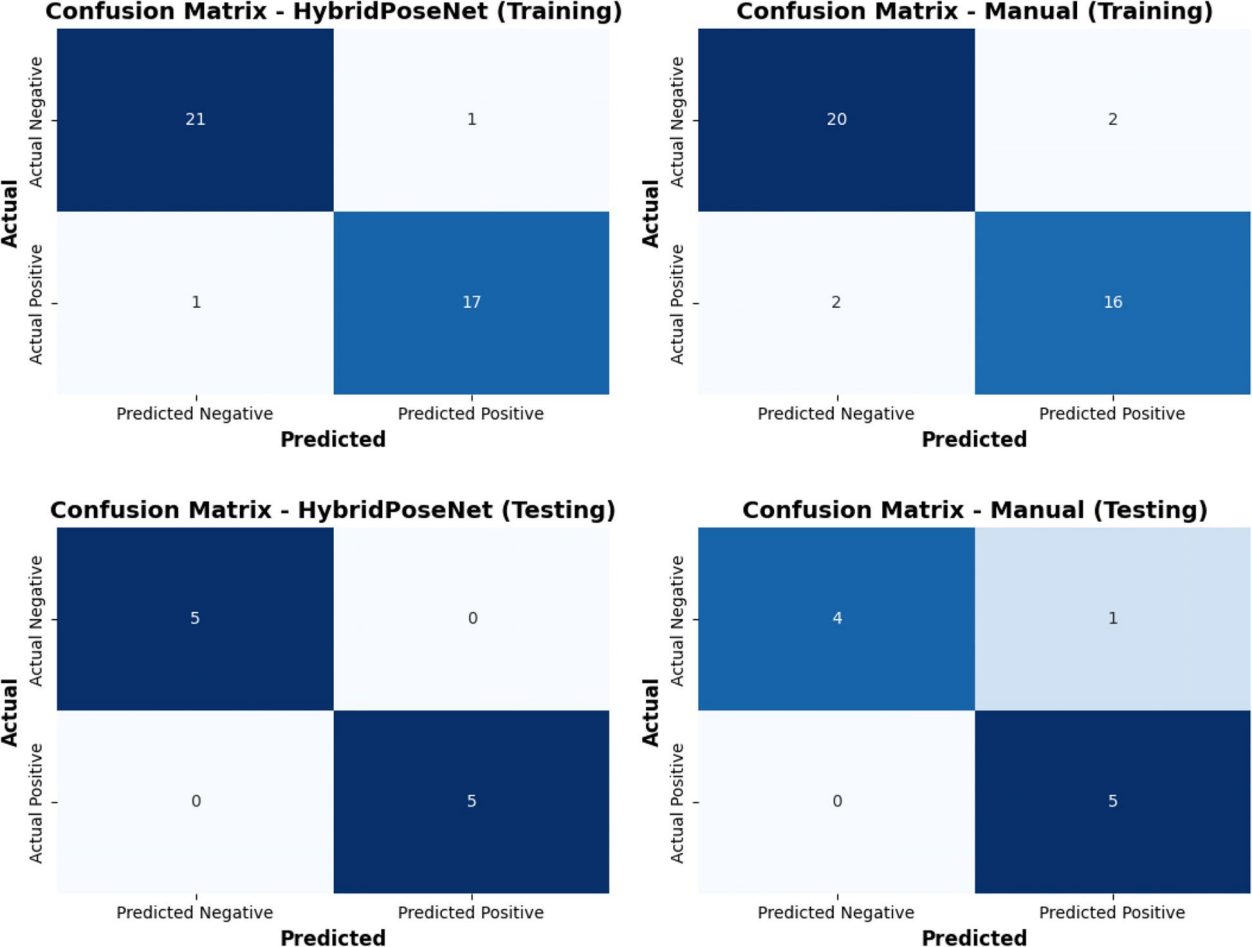
Confusion matrix assessments for HybridPoseNet and manual measurements in the diagnosis of EDS

Figure 8 shows the HybridPoseNet’s confusion matrices and manual measurements throughout the training and testing phases, showing the model’s effectiveness and the manual method’s accuracy in identifying EDS. Manual measurement in the training phase shows that the confusion matrix has 20 TN, 2 FP, 2 FN, and 16 TP, giving an accuracy of 90. However, the higher FP and FN values suggest lower precision and recall than HybridPoseNet, which may be caused by variability or manual measurement errors. This implies caution when relying solely on manual measurements to diagnose EDS. The confusion matrix of the manual measurement in the testing phase is 4 TN, 1 FP, 0 FN, and 5 TP, with a testing accuracy of 90%. The one FP in manual testing means a slight decrease in precision, which is characteristic of manual methods as they may involve human errors. For the confusion matrix of HybridPoseNet in the training phase, there are 21 TN, 1 FP, 1 FN, and 17 TP, with an accuracy of 95%. The low number of FP and FN shows that the proposed model is exact and has a high recall. The confusion matrix of HybridPoseNet in the testing phase shows 5 TN, 0 FP, 0 FN and 5 TP, which indicates 100% accuracy. The perfect classification in the testing phase shows that the proposed model is reliable for diagnosing EDS. HybridPoseNet consistently outperforms manual measurements in training and testing sets, demonstrating superior precision and recall. This analysis underscores the significant reduction in errors with the automated measurements of the model for diagnosing EDS, compared to the manual measurements, which exhibit more variability and lower reliability due to the high number of FP and FN.

The importance of this work is rooted in its attempts to encompass the problem of diagnosing the extent of EDS, especially its hypermobility subtypes, more effectively. The work from [11, 12] also stressed on the issues underlining the differential diagnosis of EDS from other features and genetic studies. However, HybridPoseNet fills this gap by giving a strong image-based goniometer that uses deep learning methods, where MobileNet-V2 is used for the spatial feature or HyperLSTM is used for temporal sequence determining and high joint angle estimation. Unlike other poses where one has to use several libraries for different joints, HybridPoseNet delivers a consolidated method for all joints to improve the diagnostics’ precision and reliability. It has high relevance to the current body of knowledge as it incorporates higher-level assessments with functional clinical correlations that allow for enhancing assessment approaches, accuracy, and standardization of joint hypermobility elements in EDS patients. HybridPoseNet’s capability of delivering constant, accurate measurements of physical changes within the body can help redress a lack of investment in the current diagnostic solutions, thereafter enhancing the clinicians’ ability to make informed decisions and design appropriate treatment plans for patients.

## Conclusion and future direction

The suggested image-based goniometry system called HybridPoseNet, built with MobileNet-V2 and HyperLSTM units, significantly advances diagnosing the EDS by providing precise joint angle estimations. The presented model, utilizing hybrid DL techniques and showing better generalization and time-dependency, can be used to properly and efficiently assess joint hypermobility. When compared to the goniometric measurements taken by the human operator, the correlation coefficients were found to be positive and highly significant for the thumb (rho = 0. 847), elbows (rho = 0. 822), knees (rho = 0. 839) and fifth fingers (rho = 0. 896) which justifies the reliability and accuracy of the device for various joints. These correlation coefficients and the results of HybridPoseNet indicate a 20% improvement over the traditional pose estimation, thus making HybridPoseNet a good candidate for standardized EDS evaluation.

With the successful application of the HybridPoseNet system in EDS diagnosis, there is a promising opportunity to extend its use to other clinical disorders. By fine-tuning the model with a larger and more diverse dataset, we plan to enhance its accuracy and generalization ability. Additionally, optimizing the feedback speed and constructing user-friendly interfaces could significantly improve the system’s clinical usefulness, offering a hopeful prospect for future advancements in medical technology.

## Data Availability

The datasets used for the findings are publicly available at https://github.com/kmkrphd/Ehlers-Danlos-Syndrome-EDS-_Video_Frame_datasets.git.
